# RNA Structure Coordinates Translation Across the Meiotic Program

**DOI:** 10.64898/2026.01.15.699792

**Published:** 2026-01-22

**Authors:** Hao Wu, Caini Zhou, Shoucheng Du, Simon Felder, Yihan Xiong, Kevin M. Weeks, Yun Bai

**Affiliations:** 1 School of Life Science and Technology, ShanghaiTech University, 201210 Shanghai, China; 2 Department of Chemistry, University of North Carolina, Chapel Hill, NC 27599-3290, USA; 3 Deceased

**Keywords:** RNA structure, meiosis, translation timing, RNA helicase

## Abstract

mRNA structure is a key determinant of translation efficiency for individual transcripts, yet its role in coordinating complex physiological processes remains elusive. We profiled mRNA structures during yeast meiosis, deriving a high-resolution structurome covering ~70% of annotated mRNAs, with multi-time-point measurements for 2,084 mRNAs. Transcripts upregulated during meiosis generally have flexible structures that enhance translation. In contrast, complex structures impede translation during meiosis by reducing ribosome flux in coding regions and promoting alternative initiation, including in the highly abundant transcript *CCW22*. We uncovered a high-low-high oscillation in cytoplasmic RNA helicase levels, which dynamically reprograms cell-wide translational preferences for RNA structure, shaping the temporal translation of hundreds of mRNAs. Disrupting RNA structure in *CCW22* or altering Ded1p helicase levels interfered with meiotic proteostasis, hindering meiosis progression. Our study reveals that the concerted action of RNA structure and RNA helicases coordinates cell-wide translation dynamics, highlighting a potent post-transcriptional regulatory layer during meiosis when transcription is limited by chromosome condensation.

## INTRODUCTION

RNA molecules adopt elaborate structures that underlie diverse regulatory functions^1^. Advances in high-throughput structure-probing have revealed RNA structural landscapes in diverse entities, from viruses to animals^2–7^. Pioneering studies have advanced our understanding of RNA folding under static conditions and emphasized broad switch-like functions for RNA structure in modulating translation. RNA structures have been evaluated within dynamic cellular contexts, such as isolated cellular compartments^8^, different phases of zebrafish embryogenesis^9^, and pattern-triggered immunity in *Arabidopsis*^10^. These studies have identified specific structural features and their roles in state-specific gene regulation. Unexplored are general rules for how RNA folding systematically coordinates gene expression during dynamic cellular processes.

RNA structure profoundly impacts translation, a process inherently dependent on mRNA-ribosome interactions. Although the majority of ribosomes, likely over 90% in bacterial cells^11^, are actively engaged in translation, a substantial fraction of mRNAs remains untranslated^12^. These observations imply that ribosome resources are scarce relative to mRNAs and emphasize the need for their stringent allocation. Moreover, the proportion of actively translated mRNAs varies across cell types and physiological states^13^, raising a critical question: What mechanisms, during dynamic processes, prioritize and alter mRNA access to limited ribosome resources?

Meiosis, a conserved eukaryotic developmental process, has been extensively studied in *Saccharomyces cerevisiae*, providing insights into the multi-layered regulation of stage-specific gene expression. Earlier studies have primarily focused on the cascade of transcription factors^14^, yet transcriptional regulation is likely limited when chromosomes are in various stages of movement and condensation, a defining feature of meiosis. Ribosome profiling studies highlight extensive translation control during meiosis^15^, involving modulation of RNA-binding protein interactions^16^, use of upstream open reading frames (uORFs)^17^, and transcript isoform switching^18^. However, the mRNA structural landscape during meiosis remains uncharacterized, leaving the regulatory roles of RNA folding unexplored. The unique attributes of budding yeast, including reduced cellular heterogeneity relative to multicellular organisms, a genome with fewer introns, and established synchronization protocols^19^, enable high-resolution determination of RNA structure during this dynamic process.

In this study, we conducted dimethyl sulfate (DMS) mutational profiling (DMS-MaP) and RNA-seq across seven meiotic stages spanning the two nuclear divisions, together with two control states of vegetative growth and pre-meiotic stage. We found that transcripts upregulated during meiosis adopted more flexible structures than those from vegetative growth. RNA structural complexity and translation efficiency (TE) showed stronger correlations during meiosis, with complex structures reducing ribosome flux in coding regions and promoting translation from uORFs. This translational preference for RNA structure displayed an oscillating pattern that delayed translation of structured mRNAs to later stages, particularly for abundant transcripts like *CCW22*. We validated the role of RNA structural elements in governing *CCW22* translation timing using data-directed structure modeling and in-cell reporter systems.

Notably, cytoplasmic mRNA helicases also showed oscillating expression patterns matching the translational preference for RNA structure. Overexpression of Ded1p helicase disrupted the timely translation of transcripts upregulated during meiosis in an RNA structure-dependent manner, causing meiosis progression to pause at metaphase II. Collectively, our work reveals how intrinsic RNA structures and oscillating helicase levels coordinate cell-wide translation timing and meiotic proteostasis. This mechanism is particularly crucial during meiosis, when gene regulation at the transcriptional level is compromised by chromosome condensation and movement, necessitating an additional layer of gene regulation at the post-transcriptional level.

## RESULTS

### High-resolution DMS-MaP Resolves mRNA Structures Across Yeast Meiosis

**Figure 1.**
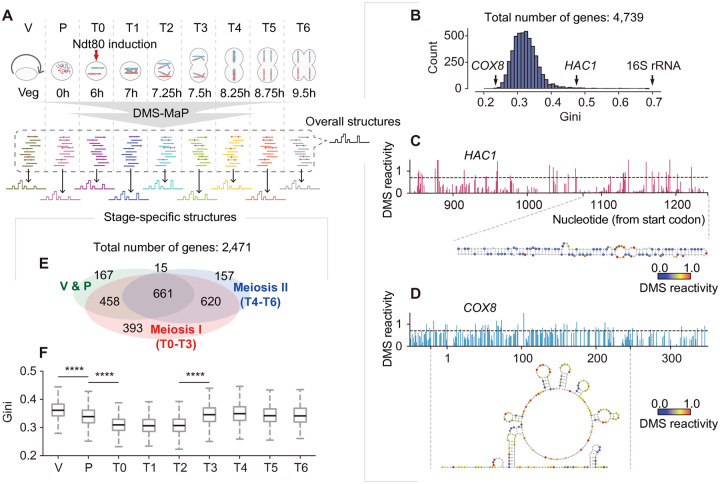
High-resolution mRNA structurome during yeast meiosis (A) Experimental strategy to obtain mRNA structural maps during yeast meiosis. (B) Gini index distribution of mRNA structures from 4,739 genes. (C) DMS reactivities of a 400-nt region in *HAC1* mRNA and corresponding structure model. (D) DMS reactivities of a 400-nt region in *COX8* mRNA and corresponding structure model. (E) Venn diagram of the numbers and relationships of stage-specific structures resolved during three periods: vegetative to pre-induction (V-P), early meiosis (T0-T3), and late meiosis (T4-T6). (F) Gini index distribution of stage-specific structures along the meiosis progression, with statistical significance noted for transitions between stages: V-P, *P* < 10^−44^. P-T0, *P* < 10^−107^. T2-T3, *P* < 10^−130^.

To generate comprehensive mRNA structure profiles of the yeast meiotic program, we employed synchronous meiosis to achieve stage-specific resolution and sampled multiple time points for optimal transcriptome coverage. Specifically, meiosis was synchronized by inducing expression of the transcription factor Ndt80p with estrogen^19^, and samples were collected across seven meiotic stages, staged by spindle morphology imaging^20^ (Fig. S1A): pachytene/pre-induction (T0), end of prophase I (T1), metaphase I (T2), anaphase I (T3), metaphase II (T4), anaphase II (T5), and meiotic exit (T6). In addition, vegetative growth (V) and pre-meiotic (P) samples were included as control stages. All samples were analyzed using mRNA-seq and in-cell DMS-MaP (Fig. 1A).

DMS-MaP measures the chemical accessibility of RNA to DMS, a reagent that preferentially modifies unpaired adenosines and cytidines. We generated a comprehensive DMS-MaP dataset for the *S. cerevisiae* transcriptome, comparable in library size (~180 million reads per stage, Table S1) to datasets obtained in studies of mammalian cells^8,21^. DMS treatment selectively modified adenosine and cytidine residues, as expected (Fig. S1B). We quantified DMS reactivities at single-nucleotide resolution^22^. Assessment of Pearson’s r value for DMS reactivities between replicates revealed that a read-depth threshold of 350 was sufficient for reproducible structure modeling (Fig. S1C), which we applied in subsequent analyses. The DMS-MaP data showed excellent agreement with structures of well-characterized RNAs like the 5S rRNA^23^ and *GLN1* mRNA^5^ (Fig. S1D).

We performed quantitative structural analysis using two approaches. First, we pooled data across all stages to generate high-quality structures for 4,739 of 6,666 annotated yeast mRNAs (Table S2), with a median signal-to-noise ratio of 5.8 (Fig. S1E). Of these, 3,399 had near-complete coverage (>80%), and 1,340 covered more than 200 A/C residues (Fig. S1F). To quantify structural complexity, we calculated the Gini index^5^, where higher values indicate more extensive DMS protection, as seen in highly structured RNAs such as rRNAs (Fig. 1B). Structural complexity varied greatly among mRNA species (Fig. 1B). For example, *HAC1* mRNA, previously shown to form extensive intramolecular base-pairings^24,25^, had a high Gini index of 0.47 (Fig. 1C), whereas *COX8* mRNA, predicted *in silico* to be largely single-stranded, had a Gini index of 0.24 (Fig. 1D).

We also resolved stage-specific structures for 2,471 mRNAs (Fig. 1E), with 2,084 spanning multiple stages, and 1,799 covering three or more stages (Table S3). The Gini index distribution across stages initially decreased and then increased (Fig. 1F). Specifically, the mean value for all structures per stage decreased from vegetative growth to pachytene (V-P: *P* < 10^−44^, P-T0: *P* < 10^−107^) and remained low until metaphase I (T2), before increasing substantially at anaphase I (T2-T3: *P* < 10^−130^) and then remaining at this level until meiotic exit (T5). This pattern suggests the presence of specific cellular factors or processes that contribute to the reduction of global mRNA structural complexity at meiotic entry.

### Transcripts Upregulated During Meiosis Adopt Simpler Folding Than Other mRNAs

**Figure 2.**
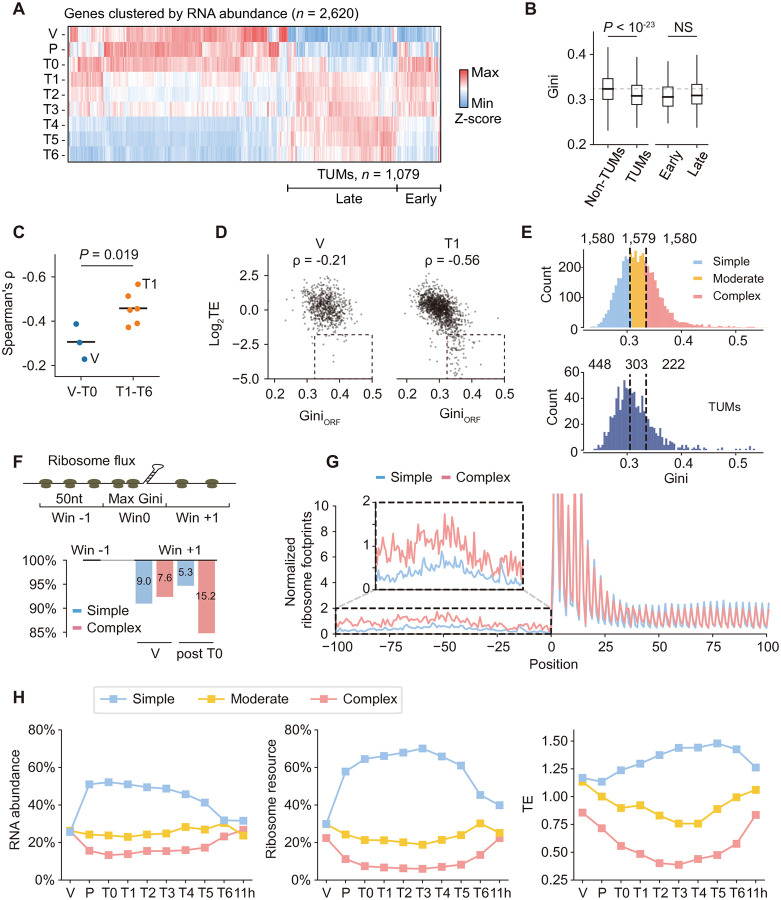
Meiotic preference for transcribing and translating mRNAs with flexible folding (A) Hierarchical clustering of RNA abundances for genes (*n* = 2,620) with more than an 8-fold change (*P* < 0.05) in transcripts per million (TPM) during meiosis. (B) Distribution of Gini indices in mRNA groups: non-TUMs (*n* = 3,767), TUMs (*n* = 937), Early TUMs (*n* = 251), and Late TUMs (*n* = 705). The dashed line indicates the median Gini index for the non-TUMs group. (C) Spearman’s rank correlation coefficients between Gini indices and TEs during V-T0 and T1-T6 periods. (D) Relationship between Gini indices and TEs at stage V (with the lowest correlation, *n* = 736) and stage T1 (with the highest correlation, *n* = 1,480). (E) Three mRNA groups were classified based on RNA structural complexity: Simple, Moderate, and Complex. The number of TUMs within each group is also indicated with corresponding cutoffs. (F) ORF ribosome flux analysis. Ribosome flux was defined as the sum of ribosome footprint reads (A site) within each 50-nt window. The window with the maximal Gini index was designated as window 0. Ribosome flux in the upstream window (−1) was used for scaling (to 100%) for comparison. (G) Ribosome footprints on uORFs. For each gene, ribosome footprints (A site) from −100 to +900 positions relative to the start codon were normalized to a total of 1,000. For each group, the distribution of ribosome footprints was averaged by position. (H) Proportional contribution of mRNA abundance and ribosome footprints. RNA abundance was scaled to 100% to measure the proportion for each group. Similarly, ribosome footprints were normalized to indicate the ribosome resource occupancy for each group. The overall translation efficiency for each group was measured by normalizing ribosome resource occupancy to mRNA abundance proportion.

Given the complex transcriptional cascade during meiosis^14,26^, we asked whether transcriptome remodeling at meiotic entry influences global mRNA structural complexity. We analyzed mRNA-seq data from the nine stages and performed hierarchical clustering of the 2,620 mRNAs that exhibited at least an eight-fold change in abundance, classifying them as either transcripts upregulated during meiosis (TUMs) or non-TUMs (Fig. 2A). Specifically, 1,078 transcripts, characterized by low vegetative expression but increased expression from pachytene (T0), were categorized as TUMs. The remaining 5520 transcripts, which did not significantly change or were downregulated during meiosis, were labeled as non-TUMs. TUMs can be further sub-categorized as early and late groups based on their peak abundance around meiosis I or meiosis II, respectively (Fig. 2A).

We found that TUMs had significantly lower Gini indices than non-TUMs (*P* < 10^−23^, Fig. 2B), and no significant Gini differences were noted between early and late subgroups of TUMs (*P* = 0.061, Fig. 2B). Thus, as cells enter meiosis, the downregulation of more structured non-TUMs and the upregulation of less structured TUMs collectively reduce global mRNA structural complexity. The subsequent downregulation of early-group TUMs in later meiotic stages may account for the recovery of global structural complexity (Fig. 1F).

Because GC content of an mRNA can, in principle, influence its RNA structural complexity, we asked whether G–C rich codons contribute to the overall flexible folding of TUMs. Comparison of GC content within open reading frame (ORF) regions revealed no significant difference between TUMs and non-TUMs (*P* = 0.4, Fig. S2A). To further exclude the impact of nucleotide composition on structural quantification, we calculated Gini index using only adenosine reactivities. TUMs retained a significantly higher Gini(A) index, indicating reduced base-pairing of adenosines, independent of G or C nucleotides (Fig. S2B). Together, these results demonstrate that the structural differences between TUMs and non-TUMs are not driven by simple differences in nucleotide composition, but instead reflect intrinsic differences in mRNA folding. Given that RNA structures can impede translation and often require ATP-dependent unwinding, the reduced base-pairing in TUMs may reflect evolutionary selection under nutrient-limiting conditions, potentially facilitating more efficient protein synthesis during meiosis.

### Coupled Preference for RNA Structure at Transcription and Translation Levels

A prior ribosome profiling study revealed widespread translation control throughout yeast meiosis^15^. Translation efficiencies (TEs) of certain mRNAs like *SPS1* varied up to 100-fold^15^. Although active translation is known to destabilize mRNA structures within coding regions, the extent of this effect in eukaryotes is unclear. We asked whether the simpler folding of TUMs is shaped by translation regulation. Using matched time courses from ribosome profiling and our structurome datasets (Fig. S2C), we examined RNA structural changes in transcripts with dramatic TE fluctuations, including *ECM8*, *SPO20*, *SPS1*, *CLB3*, and *GIP1*^15^. Surprisingly, DMS reactivities remained highly preserved despite TE variations ranging from 15- to 106-fold (Fig. S2D). Analysis of ORF structural complexity (Gini_ORF_) in 390 transcripts with at least 4-fold TE changes showed no correlation between complexity and TE changes (r = −0.08; *P* = 0.16, Fig. S2E). These results suggest that the ORF structures are preserved in highly translated mRNAs, potentially due to rapid RNA refolding after ribosome passage. The refolded structures could hinder subsequent ribosomes, highlighting the potential for ORF structure-mediated translation regulation.

We then investigated how preserved ORF structures influence translation by examining the genome-wide association between TE and Gini_ORF_. Complex ORF structures are postulated to hinder translation elongation^27^. Consistent with this, we observed modest negative correlations between Gini_ORF_ and TEs across all vegetative and meiotic stages (Spearman’s r: −0.32, *P* < 10^−261^, Fig. S2F). The correlations were notably stronger during meiotic stages when TUMs are fully transcribed (T1-T6, Spearman’s r: −0.35 ~ −0.56) compared to stages of vegetative growth to pachytene (V-T0, Spearman’s r: −0.21 ~ −0.36, Fig. S2G). The strongest correlation emerged at the end of prophase I (T1, Spearman’s r = −0.56), where mRNAs with high Gini indices showed significantly lower TEs (Fig. 2D), suggesting enhanced translational repression of highly structured mRNAs during meiosis. Correspondingly, the correlation between RNA abundance and ribosome profiling weakened significantly during meiotic stages (Fig. S2H), further supporting RNA structure as a dominant regulator of translation during meiosis.

To validate the association between RNA structure and TE, we stratified transcripts with quantified structures into three equal-sized groups based on overall structural complexity: Simple, Moderate, and Complex (Fig. 2E). Among TUMs, the Simple group (*n* = 448) contained twice as many transcripts as the Complex group (*n* = 222), consistent with a general trend toward simpler folding (Fig. 2E). Transcripts in the Simple group exhibited significantly higher TEs at all stages, with this difference more pronounced during meiosis: average TEs for Simple group increased from ~1.4-fold during vegetative growth to more than 2.0-fold during meiosis compared to Complex group (Fig. S2I). Consistent with this trend, TUMs displayed relatively low TEs during vegetative growth but achieved higher TEs during meiosis, particularly at early stages (T2 and T3, Fig. S2J), indicating that the meiotic cellular environment is especially permissive for TUM translation. Together, these results demonstrate that the regulatory impact of RNA structure on translation is substantially amplified during meiosis.

To elucidate the mechanisms underlying the amplified translational divergence, we confirmed that structural differences between the Simple and Complex groups were maintained across the 5′UTR, ORF, and 3′UTR regions, where mRNAs in the Complex group exhibited consistently lower DMS reactivities (Fig. S2K). To assess how local ORF structures influence ribosome movement, we segmented ORFs into 50-nucleotide windows and calculated ribosome flux for each window (Fig. 2F). Ribosome flux declined progressively from the 5′ to the 3′ end (Fig. S2L). We defined the most structured region (window 0) as the window with maximal Gini index, which was significantly higher in the Complex group (Fig. S2M), reflecting more intricate local RNA structures. During vegetative growth, ribosome flux decreased similarly in both groups when comparing upstream (−1) and downstream (+1) windows, with a minimal difference of 1.4%. In contrast, during meiosis, the Complex group exhibited ~10% greater reduction in ribosome flux (Fig. 2F), indicating that local RNA structures impose stronger barriers to ribosome progression during meiosis. Given that translation from upstream open reading frames (uORFs) in yeast occurs predominantly during meiosis^15^, we asked whether 5′UTR structure influences uORF translation. Analysis of ribosome footprinting in 5′UTRs revealed that the Complex group exhibited twice the frequency of uORF translation compared to the Simple group (Fig. 2G), suggesting that 5′UTR structure promotes alternative start codon usage and enhances uORF translation, thereby suppressing translation of the downstream main ORF.

Overall, our results reveal a meiotic preference for transcription and translation of mRNAs with flexible folding. To quantify this preference, we scaled RNA abundance and ribosome footprint signals to 100% at each time point and calculated the proportional contributions of transcripts stratified by structural complexity. This analysis uncovered strong coupling among transcriptome composition, ribosome resource allocation, and RNA structure (Fig. 2H). Notably, both RNA abundance and translation displayed an oscillatory preference for RNA structure. During vegetative growth, mRNAs of differing structural complexity contributed comparably. Upon entering meiosis, however, transcripts with flexible folding became overrepresented and were preferentially engaged by ribosomes, before returning to a more balanced distribution at meiotic exit (Fig. 2H). To further define translational preferences independent of RNA abundance, we normalized the ribosome occupancy by mRNA abundance, revealing a similar oscillation pattern in TE across meiosis (Fig. 2H). These findings demonstrate dynamic coupling between transcriptional output and translational prioritization based on RNA structure, enabling efficient ribosome resource allocation to newly transcribed mRNAs during meiosis.

### mRNA Structure Contributes to Stage-Specific Gene Expression

**Figure 3.**
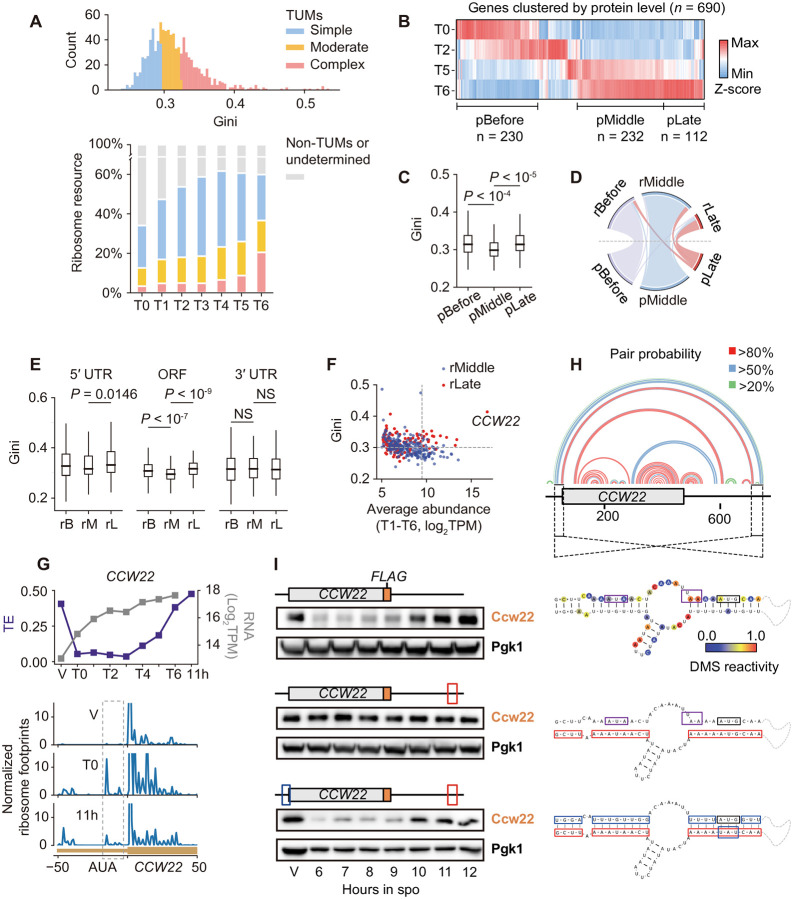
mRNA structure differences regulate translation timing (A) Distribution of Gini indices for TUMs (upper) and dynamic ribosome resource allocation (lower) for three mRNA groups classified based on structural complexity: Simple (*n* = 324), Moderate (*n* = 325), and Complex (*n* = 324). (B) Hierarchical clustering of protein abundances for genes exhibiting more than 1.2-fold changes (*n* = 690, *P* < 0.05). (C) Comparison of Gini indices among three protein groups: before meiosis (pBefore, *n* = 192), during mid-meiosis (pMiddle, *n* = 223), and near meiotic exit (pLate, *n* = 104). (D) Overlap analysis between genes in ribosome profiling and protein clusters: Before, *P* < 10^−38^; Middle, *P* < 10^−50^; Late, *P* < 10^−18^. (E) Comparison of Gini indices among ribosomal profiling clusters (rB, rBefore; rM, rMiddle; rL, rLate) across the 5′UTR (rB, *n* = 331; rM, *n* = 184; rL, *n* = 121), ORF (rB, *n* = 930; rM, *n* = 268; rL, *n* = 188), and 3′UTR (rB, *n* = 751; rM, *n* = 227; rL, *n* = 143). Only UTRs with coverage of more than 20 A/C were included. Note that the smaller sample size in the 5′UTR contributes to a larger p-value compared to the ORF, which does not necessarily indicate a weaker regulatory effect. (F) Relationship between Gini indices and average mRNA abundance (log_2_TPM) during meiotic stages T1-T6. (G) *CCW22* RNA abundance and TE changes during meiosis (upper), and ribosome footprints on *CCW22* uORF at high (V and11h) and low TE (T0) stages (lower). The ribosome footprint reads were normalized similarly as in Fig. 2G (lower panel). (H) Data-directed structure model of *CCW22* mRNA, shown as an arc plot of base-pairing probabilities. (I) Western blot analysis of Ccw22p expressed from wild-type (top), structure-disrupted (middle), and structure-restored (bottom) vectors. The red box indicates nucleotides mutated for structural disruption. The blue box indicates nucleotides mutated to restore the 5′-end base pairing with the 3′UTR. The purple boxes indicate the start codon and stop codon of a *CCW22* uORF. Note that the compensatory mutations in the 5′UTR also alter the Kozak sequence. While an oscillatory expression pattern is restored, the Ccw22p expression strength could be affected.

A central question in meiosis is how pre-transcribed mRNAs are selectively translated at distinct stages. Although TUMs generally have simpler structures, their structural complexity still varies considerably (Fig. 3A). We explored whether RNA structure differences among TUMs influence their translational dynamics. We similarly classified TUMs into three equal groups based on Gini indices and compared their ribosome resource occupancy from pachytene (T0). The Simple group peaked in ribosome resource occupancy around anaphase I (T3: 40%), while the Complex group surged only during meiotic exit (T5: 8.7%, T6: 20.5%). The Moderate group had a pattern that fell between these two extremes (Fig. 3A). This pattern is strongly associated with the structural complexity in 5ʹUTR and ORF regions, but not 3ʹUTR regions (Fig. S3A). Gene Ontology (GO) analysis revealed stage-specific functional associations: Genes involved in early-stage events such as synapsis, meiotic recombination, and chromosome segregation were predominantly in the Simple and Moderate groups. In contrast, genes in the Complex group were primarily associated with cell wall activities (Fig. S3B and Table S4), which are critical during gamete maturation following the two meiotic divisions. Moreover, genes known for translation delayed until the end of meiosis II^28^, such as *SPS1*, *SPS4*, and *SSP2*, were significantly enriched in the Complex group (*P* < 10^−7^, Table S5). These findings suggest that ribosome resources are dynamically allocated to TUMs in an RNA structure-dependent manner to support their stage-specific functions.

To evaluate the role of RNA structure in meiotic protein dynamics, we conducted quantitative proteomics across four meiotic stages (Table S2): pachytene (T0), metaphase I (T2), anaphase II (T5), and meiotic exit (T6). Among 3,989 quantified proteins, including both TUMs and non-TUMs, 690 changed significantly (>1.2-fold, *P* < 0.05). These proteins clustered into three groups based on their peak abundance: before meiosis, during mid-meiosis, and near meiotic exit (Fig. 3B). Proteins peaking in mid-meiosis had significantly simpler RNA structures compared to the other groups (Fig. 3C), aligning with the oscillatory preference for RNA structure. These results confirmed a connection between mRNA structure and meiotic protein dynamics.

To evaluate the role of RNA structure in translation timing, we classified 1,666 genes with >8-fold changes in ribosome footprints into three groups, similar to those in protein clusters, but based on their translation profiles (Fig. S3C). Significant overlaps in mRNAs clustered by ribosome footprint or protein level at each stage (before, *P* < 10^−38^; middle, *P* < 10^−50^; late, *P* < 10^−18^; Fig. 3D) indicate that translation timing governs meiotic protein dynamics. Consistent with the trend in proteins, highly translated mRNAs exhibited high-low-high oscillation in structural complexity (Fig. S3D). Notably, 217 transcripts with peak translation near meiotic exit, including Ndt80 targets known for delayed translation timing such as *CLB3*^19^ and *SPS1*^28^, had more complex structures than mRNAs translated during middle meiosis (*P* < 10^−11^), and were enriched in the Complex group (*P* < 10^−7^, Table S6). RNA-seq profiles revealed that these genes were transcribed during early stages, from pachytene to metaphase II (Fig. S3E), highlighting varying degrees of translation delays. Gini index comparison revealed that mRNAs with peaked translation near meiotic exit had higher complexities in 5ʹUTRs and ORF (*P* = 0.016 and *P* < 10^−9^, respectively), but not 3ʹUTRs (Fig. 3E and Table S7), suggesting that complex 5ʹUTR and ORF structures delay translation.

Given that abundant transcripts impose greater demands on ribosome resources, we analyzed RNA abundance among mRNAs translated during middle and late meiosis and found that highly abundant transcripts exhibit pronounced structural differences associated with translational timing (Fig. 3F). For example, *CCW22*, the most abundant mRNA during meiosis encoding a cell wall protein, was translated near meiotic exit and had an exceptionally high Gini of 0.414 (Fig. 3F). Despite continuous accumulation, *CCW22* mRNA showed relatively high TEs only during vegetative growth and again during late meiotic stages (Fig. 3G). Ribosome footprint analysis revealed increased use of a near-cognate start codon (*AUA*) at position −17 in its 5′UTR specifically during low TE stages (Fig. 3G). This codon resides in a long-range 22 bp interaction spanning the 5′ and 3′UTR, as revealed by DMS-MaP structural modeling (Fig. 3H). The stem harboring the *AUA* codon shows lower pairing probability, suggesting ribosome stalling here may locally disrupt the structure (Fig. 3H). The 5′UTR-3′UTR interactions appear to hinder ribosome progression during specific stages, thereby promoting near-cognate start codon usage.

To confirm a role for the 5′UTR-3′UTR interactions in regulating *CCW22* translation timing, we truncated either UTR in a reporter construct, which led to earlier Ccw22p expression, compared to the wild-type construct (Fig. S3F). Disrupting the interactions by mutating 3′UTR nucleotides that pair with the 5′UTR similarly resulted in earlier Ccw22p expression (Fig. 3I). Restoring the interactions by mutating the 5′UTR rescued the oscillatory expression (Fig. 3I). All constructs showed rapid mRNA accumulation during early stages (Fig. S3G), but structure-disrupted mRNAs failed to further accumulate at late stages, suggesting that 5ʹUTR-3ʹUTR interactions also influence mRNA stability. Despite the greater accumulation of RNA abundance from wild-type and structure-restored constructs, protein synthesis is largely suppressed during early meiotic stages. These results validate that the long-range 5′UTR-3′UTR interactions of *CCW22* modulate its oscillatory translation.

Consistent with its extreme abundance, ribosome resources occupied by *CCW22* increased from 0.05% during pachytene to 4.2% at meiotic exit (Fig. S3H). This pronounced engagement underscores the capacity of structural elements within *CCW22* to influence global translation events. Supporting this model, a yeast strain expressing a *CCW22* variant unable to form 5′UTR-3′UTR interactions showed a significant delay in meiosis progression (Fig. S3I), despite *CCW22* not having been previously implicated in meiosis. Together, these results show that failure to restrain translation of highly abundant transcripts can exert cell-wide effects through competitive access to a finite ribosome pool, underscoring the role of RNA structure in ensuring proper meiotic progression.

### mRNA Unwinding Power Oscillates to Coordinate Meiotic Proteostasis

We hypothesized that the dynamic allocation of ribosome resources arises from fluctuations in cellular sensitivity to mRNA structures, or what we term mRNA unwinding power. RNA helicases have been identified as the primary factors unwinding RNA structures *in vivo*^29^. In mammals, RNA helicases such as YTHDC2, DHX36, and Ddx25 play pivotal roles at various stages of meiosis and spermatogenesis^30–32^, suggesting that these factors precisely modulate unwinding power across different developmental stages.

**Figure 4.**
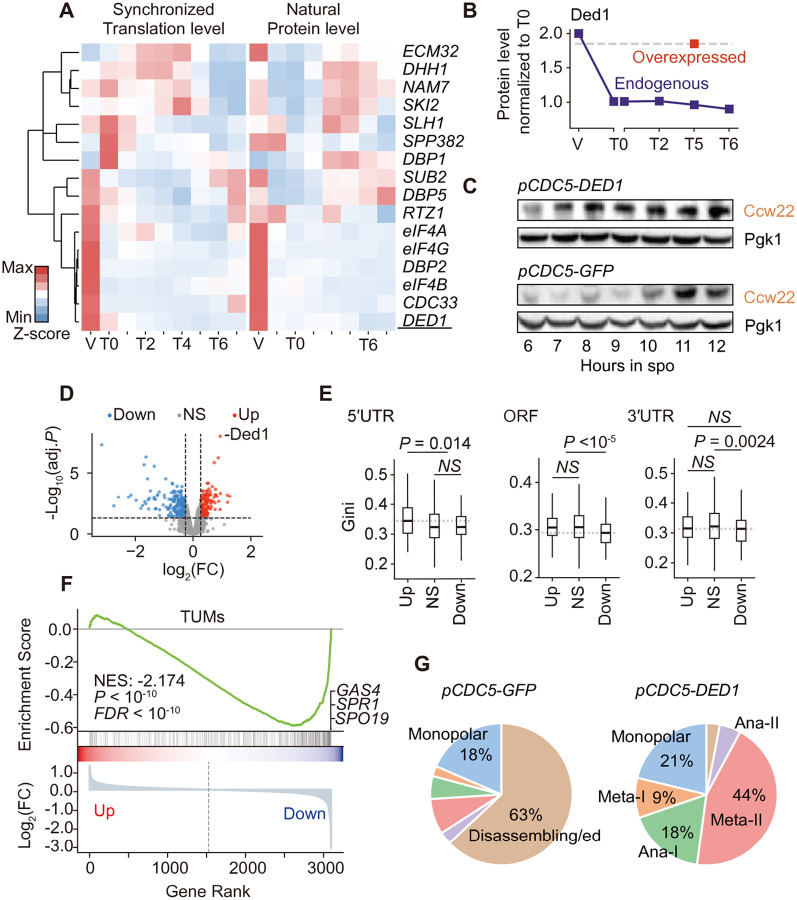
mRNA unwinding power coordinates meiotic proteostasis (A) Co-hierarchical clustering of 14 cytoplasmic RNA helicases and 2 accessory proteins during meiosis. Ribosome profiling data (left) were from synchronized meiosis, and proteomic data (right) were from strain undergoing natural meiosis. (B) Endogenous Ded1p protein levels (blue squares) and overexpressed Ded1p protein level (red squares) at T5 stage driven by the *CDC5* promoter. (C) Western blot analysis of Ccw22 expression in strains with Ded1p or GFP overexpression, both under the control of the *CDC5* promoter. (D) Volcano plot of genes differentially expressed under Ded1 overexpression, with fold change (FC) > 1.2 and P < 0.05. Upregulated, *n* = 149; Downregulated, *n* = 194; Not significant, *n* = 2,761. (E) Comparison of Gini indices across 5′UTR, ORF, and 3′UTR regions between differentially expressed genes under Ded1 overexpression. (F) Gene Set Enrichment Analysis of TUMs protein level changes under Ded1 overexpression. (G) Cell percentage of different meiotic stages at 12 hours post-induction in yeast strains overexpressing GFP or Ded1p.

To evaluate mRNA unwinding power across meiosis, we analyzed the expression of 14 cytoplasmic RNA helicases and 2 helicase-regulating accessory proteins. Because our study lacks proteomic quantification of vegetative growth and gamete maturation, we referenced a prior study of SK1 yeast undergoing natural meiosis^18^ (Table S8). Co-clustering of helicase expression at the translation and protein levels revealed two distinct patterns (Fig. 4A). One group, including Ded1p, showed a continuous decline from vegetative growth to gamete maturation, while another group, like Dbp1p, showed low levels in early meiosis, followed by an increase in later stages. Overall, mRNA unwinding power oscillates across meiosis, as shown by the high-low-high pattern of total ribosome footprints of these helicase genes (Fig. S4A).

We propose that transcripts respond differentially to oscillating unwinding power based on their intrinsic folding. Under low unwinding power, mRNAs with flexible folding are efficiently translated, whereas those with complex structures are not. Conversely, in a high-unwinding-power state, all mRNAs receive sufficient helicase-mediated unwinding for efficient translation. This model aligns with observed cellular translational preferences: mRNAs with flexible folding are preferentially translated during a low-helicase state at early meiotic stages, while mRNAs with complex folding, such as *CCW22*, are efficiently translated only in a high-helicase state, including vegetative growth and later meiotic stages (Fig. 2H and Fig. 3E).

To test this model, we disrupted meiosis-specific suppression of RNA helicases by overexpressing Ded1p, a helicase known to resolve 5′UTR structures^33^. Both ribosome profiling and proteomic data revealed a 50% decrease in Ded1p levels from vegetative growth (V) to pachytene (T0), which remained at low levels until the end of meiosis (Fig. 4B and Fig. S4B). To elevate Ded1p levels, we used the *CDC5* promoter, identified via GFP reporter assays to drive robust expression during meiosis (Fig. S4C). Quantitative proteomic analysis revealed that Ded1p rose to a near-vegetative level by anaphase II (T5, Fig. 4B) under control of *CDC5* promoter. Given that repressed Ded1p enhances the use of near-cognate translation initiation codons^17^, we measured Ccw22p abundance under Ded1p overexpression and observed premature Ccw22p expression starting from early meiosis I (Fig. 4C). This result supports that elevated Ded1p alleviated ribosome stalling and alternative initiation at the near-cognate start codon within the 5′UTR-3′UTR structure (Fig. 3H), thereby releasing its translation.

Disrupted mRNA unwinding power should lead to misallocation of global ribosome resources in an RNA structure-dependent manner, thereby disrupting meiotic proteostasis. To test this prediction, we conducted differential protein expression analysis comparing wild-type and Ded1p-overexpressing conditions. This comparison identified 149 genes upregulated and 194 genes downregulated by at least 1.2-fold under Ded1p overexpression (*P* < 0.05; Fig. 4D and Table S9). Significantly, upregulated genes had more complex mRNA structures than downregulated ones (*P* < 10^−5^; Fig. S4D). Region-specific comparisons showed that upregulated genes had more structured 5′UTRs (*P* = 0.014, Fig. 4E), consistent with Ded1p’s role in unwinding 5′UTR secondary structures. In contrast, downregulated genes showed simpler ORF structures (*P* < 10^−5^, Fig. 4E), suggesting that their suppression may arise indirectly from ribosome reallocation rather than direct Ded1p effects. The 3′UTRs of downregulated genes were also less structured than genes with non-significant changes, but the difference was subtler. These findings confirm that elevated helicase levels during meiosis globally skew translation resources toward mRNAs with complex structures.

**Figure 5.**
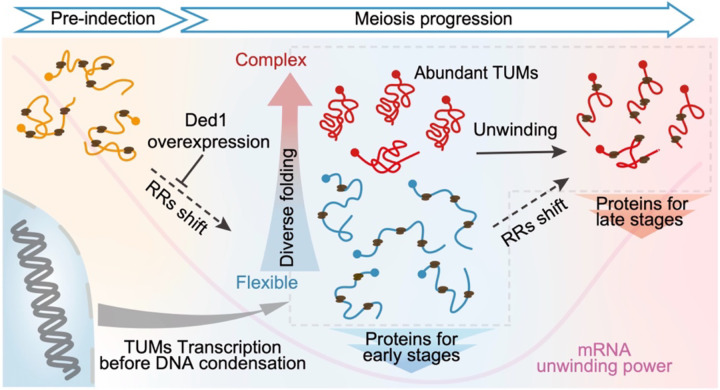
RNA structure and mRNA unwinding power orchestrate stage-specific translation Model for how diverse RNA structures, coupled with oscillating mRNA unwinding power, synergistically orchestrate the dynamic allocation of ribosome resources (RRs). These combined features enable selective translation and precise protein synthesis during meiosis.

Because TUMs generally adopt flexible structures favored during low-unwinding-power stages, we evaluated how elevated unwinding power influences TUM expression and meiotic progression. Gene Set Enrichment Analysis (GSEA) revealed a widespread decrease in abundance for TUM-encoded proteins upon Ded1p overexpression (Fig. 4F), with the strongest reductions observed for the most structurally flexible TUMs, including *SPO19*, *SPR1*, and *GAS4* (Gini = 0.28, 0.27, 0.27, respectively). Consistent with these changes, Ded1p overexpression severely disrupted the meiotic cell cycle, frequently causing arrest at or before metaphase II, as revealed by immunofluorescence staging at 12 hours post-induction (Fig. 4G). Together, these results show that fluctuations in mRNA unwinding power orchestrate ribosome resource allocation among mRNAs with differing structural complexities (Fig. 5). This mechanism directs orderly gene expression and is crucial for faithful execution of meiotic program.

## Discussion

High-throughput analysis of mRNA structure in eukaryotes requires deep sequencing to capture low-abundance transcripts within complex genomes, and extending such analyses across dynamic biological processes presents an even greater challenge. Most studies to date have focused on a few cell states or individual RNA elements, leaving a gap in our understanding of how RNA structure systematically regulates dynamic cellular processes. Here, we generated a high-resolution mRNA structural map across nine stages of synchronized yeast meiosis, covering approximately 70% of annotated mRNAs. Integrating this dataset with measurements of mRNA abundance, translation, and protein output revealed RNA folding as a broad regulatory program that coordinates selective gene expression across meiotic stages to ensure accurate execution of meiosis (Fig. 5).

Upon entering meiosis, cells transcribe a large cohort of mRNAs (TUMs) whose structures are generally flexible but still exhibit considerable diversity in their folding. During early meiosis, cytoplasmic RNA helicases are globally suppressed, limiting unwinding power and biasing ribosome resources toward mRNAs with flexible structures, including most TUMs. In contrast, highly structured mRNAs encounter translation inhibition, as complex structures reduce ORF ribosome flux and enhance uORF translation (Fig. 2F and Fig. 2G). This disparity in mRNA translatability enables selective expression for newly transcribed TUMs and facilitates rapid translational reprogramming into a meiotic mode. During late meiosis II, upregulation of specific helicases restores mRNA unwinding power, releasing translational repression of structured mRNAs. This mechanism highlights a post-transcriptional regulatory layer embedded in intrinsic mRNA folding potential, which becomes particularly critical when chromosome compaction limits new transcription during meiotic divisions.

Through time-resolved multi-omics clustering at both transcriptional (Fig. 2A and 2B) and translational (Fig. S3C and S3D) levels, we identified general RNA structural features associated with temporal regulation. For example, mRNAs with complex 5′UTR and ORF structures tend to be translated at later stages, enabling coordinated expression of functionally synergistic proteins. Similar structural features have been observed in mRNAs that participate in other environmental response processes, including double-stranded RNA structures downstream of upstream AUGs in immune response genes^10^ and structured regions that occur shortly after start codons in autophagy genes^34^. Such findings suggest that shared mRNA structural features serve as a widespread strategy to coordinate synergistic gene expression in response to complex environmental cues.

Beyond identifying functional RNA structures, our study supports a systemic model in which mRNAs compete for a finite pool of ribosome resources based on their intrinsic folding (Fig. 2H and 3A). This zero-sum model emphasizes how translation can be globally coordinated by RNA structure. For example, *CCW22*, a highly abundant transcript, adopts elaborate 5′UTR-3′UTR interactions that prevent monopolization of ribosome resources at inappropriate stages. Critically, ribosome resource allocation is dynamically modulated by RNA helicases in an RNA structure-dependent manner, such that changes in helicase expression mean that mRNA groups with similar structural complexity gain or lose a translational advantage. This framework provides a unified explanation for prior observations, including the effects of Ded1 inactivation on global TEs^35^: Ded1 inactivation suppresses translation of mRNAs with structured 5′UTRs, and simultaneously enhances translation of mRNAs with simple 5′UTRs. Focusing solely on individual transcripts makes it difficult to explain the enhanced translation under helicase inactivation. By contrast, our model attributes this dual effect to a transcriptome-wide reallocation of ribosome resources from highly structured to more flexible mRNAs, a finding supported by our helicase overexpression experiments (Fig. 4D and 4E).

We observed that mRNA unwinding power oscillates in a high-low-high pattern during meiosis, a combinatorial outcome of the expression dynamics of two classes of cytoplasmic helicases. The mRNA unwinding power described here is an estimate derived from their collective expression patterns. This approximation was used because the roles of many helicases in translation have not been thoroughly studied, and their unwinding activities are influenced by multiple factors such as accessory proteins and RNA-binding proteins^40^. We confirmed that Ded1p suppression is required for preferential translation of TUMs, particularly those with highly flexible structures (Fig. 4F). However, the helicases responsible for activating translation of highly structured mRNAs remain to be identified. Proteomic profiling of natural meiosis revealed a coordinated upregulation of several helicases known to be involved in mRNA translation (Fig. 4A). For example, Dbp1p, a ‘low performance’ paralog of Ded1p^36^, may substitute in unwinding structured 5′UTRs, while Dhh1p, associated with ORF structure unwinding^34^, may facilitate translation of structured ORFs during late meiosis. Although this study focuses on meiosis, fluctuations in helicase expression have been implicated in other physiological and pathological contexts, including innate immune responses, neurodegenerative diseases, and multiple cancers^37^. As shown here for meiosis, helicase-mediated ribosome resource reallocation at the post-transcriptional level may represent a common mechanism that regulates diverse biological processes.

## METHODS

### Synchronous meiosis conditions

The yeast strain A14201 (SK1 background), commonly used in meiotic studies, was cultured to saturation in YPD medium (1% yeast extract, 2% peptone, 2% glucose). This culture was then diluted to an optical density at 600 nm (OD_600_) of 0.3 in YPA medium (1% yeast extract, 2% peptone, 1% potassium acetate, pH = 7.0) and grown overnight. Cells were washed twice and resuspended in SPO medium (0.5% potassium acetate, pH = 7.0, 0.02% raffinose) to an OD_600_ of 1.9. At 6 hours after transfer to SPO, 1 μM β-estradiol was added to induce the meiotic transcription factor Ndt80.

### Meiotic staging

Tubulin immunofluorescence was performed to monitor spindle morphology as previously described^20^, with minor modifications. Cells were fixed with 3.7% formaldehyde for 20 minutes at room temperature, washed twice with 1x PBS, and resuspended in 100 μl of 1 M sorbitol/PBS. Cell walls were digested with 0.2 U/μl lyticase (Yeasen, 10403ES81) for 20 minutes at 30°C for vegetative cells and 30 minutes at 30°C for sporulating cells. The cells were then adhered to polylysine-treated slides and immersed in −20°C methanol for 6 minutes, followed by 30 seconds in acetone. After air drying, cells were incubated with 5% bovine serum albumin, and the yeast spindle was visualized using primary antibody Mouse anti-alpha-tubulin (Sigma, T5168, 1:1,000) and secondary antibody Cy5-conjugated goat anti-mouse IgG (Jackson ImmunoResearch Laboratories, 115-175-146, 1:200), with DAPI staining for the nuclear region.

### DMS-MaP library generation

For *in vivo* modification, 4 ml of yeast cultures (OD_600_ = 0.8 for vegetative) were treated with 200 μl DMS for 4 minutes at 30 °C. Control samples were treated with an equal amount of DMSO. The reaction was quenched with 4 ml of ice-cold stop solution (30% beta-mercaptoethanol), and cells were quickly pelleted and washed with 2 ml stop solution. Total RNA was isolated using a modified hot phenol method suitable for meiotic cells^38^ and treated with RNase-free DNase I (Promega, M6101) to remove genomic DNA. Poly(A) mRNA was enriched from 5 μg of total RNA using a Poly(A) mRNA Magnetic Isolation Module (NEB, E7490).

Reverse transcription and second-strand synthesis for mutational profiling were adapted from previous methods^21,39^, with modifications to preserve strand information. The first strand was synthesized using SuperScript II (Invitrogen, 18064014) with MaP buffer (50 mM Tris [pH = 8.0], 75 mM KCl, 6 mM MnCl_2_, 10 mM DTT, 0.5 mM dNTPs) and strand-specific reagent (NEB, E7765). Following G-25 microspin column desalting (GE Healthcare, 27532501), the cDNA was compatible with the second-strand synthesis module in the directional RNA library prep kit (NEB, E7765), which included dUTP incorporation. After purification using SPRIselect beads (NEB, E7765), the double-stranded DNA was enzymatically fragmented using the DNA fragmentation module (NEB, E7810). Most fragments were roughly 400 bp in length. Further library generation procedures, including adaptor ligation, USER enzyme digestion, size selection (> 200bp, 1:1 ratio of beads to sample), and PCR amplification, were performed as per the manufacturer’s instructions (NEB, E7765).

### Tandem Mass Tag-based LC-MS/MS

For meiotic samples, yeast cultures (2 ml) were spun down and resuspended in 0.7 ml lysis buffer (20 mM HEPES [pH = 7.5], 140 mM KCl, 2 mM EDTA, 1% Triton X-100, 1 × protease inhibitor (APExBIO, K1010)). Cells were then lysed by rigorous vortexing at 4°C with acid-washed glass beads (0.4-mm diameter). Cell debris was removed by centrifugation. The crude proteins were precipitated by adding 10 volumes of cold acetone to the supernatant and kept at −20 °C overnight. The proteins were pelleted by centrifugation and dried with the lid open. The pellet was dissolved in 100 μl urea buffer (8 M Urea, 50 mM NH_4_HCO_3_).

To disrupt disulfide bonds, dithiothreitol was added to a final concentration of 5 mM, and samples were incubated for 1 hour at 37 °C in a thermal mixer. Protein concentration was determined using a BCA assay (Yeasen, 20201ES76). Subsequently, 20 mg total protein was then alkylated with 10 mM iodoacetamide for 45 minutes at room temperature. Alkylated proteins were diluted 1:8 with 50 mM NaHCO_3_ and digested using trypsin protease (Life/Invitrogen, 90057) for 16 hours with an enzyme-to-substrate ratio of 1:50. The digestion was stopped by the addition of trifluoroacetic acid to a final concentration of 0.4%. The peptides were desalted over C18 columns and eluted in 70% acetonitrile, 0.1% formic acid. Then the samples were evaporated to dryness in a vacuum concentrator and re-dissolved in 50 μl 50 mM tetraethylammonium bromide.

Peptides were then labeled with 0.2 mg TMT10plex reagent (Thermo Scientific, 90309). The labeling reaction was stopped by adding 1 μl of 5% hydroxylamine. Samples were mixed, desalted over C18 columns, and eluted in 70% acetonitrile, 0.1% formic acid. After drying, the sample was re-dissolved in 500 μl of 50 mM tetraethylammonium bromide. Reverse-phase chromatography was used to reduce peptide complexity; 36 fractions were eluted with gradient elution buffer (increasing concentration of acetonitrile, 0.1% formic acid) and combined into 8 fractions (1–2,10,18,26; 3,11,19,27; 4,12,20,28; 5,13,21,29; 6,14,22,30; 7,15,23,31; 8,16,24,32; 9,17,25,33–36). After evaporation to dryness, liquid chromatography-tandem mass spectrometry (LC-MS/MS) analyses were performed on a Q-Exactive HF-X.

### Western blotting assay and RT-qPCR

Protein samples from yeast cultures were prepared and resolved via SDS-PAGE, followed by transfer to polyvinylidene difluoride membranes. Pgk1 and Ccw22-FLAG were detected using primary antibodies Mouse anti-PGK1 (Abcam, ab113687, 1:10,000) and Mouse anti-FLAG (Sigma, F1804–50UG, 1:2,500), respectively. Peroxidase-conjugated goat anti-mouse IgG secondary antibody (Jackson ImmunoResearch Laboratories, 115-035-146, 1:10,000) and ECL luminescent solution (Share-Bio, SB-WB012L) were used for blot detection. For RNA analysis, 2 mL samples from corresponding time points were subjected to hot phenol extraction to isolate total RNA, reverse-transcribed (Vazyme, R333–01), and quantified by RT-qPCR (Takara Bio, RR820A) using *ACT1* mRNA as an internal control.

### Gene cloning and expression in yeast

The pRS shuttle vector, containing autonomously replicating sequence and centromeric sequence, was used to leverage endogenous replication and chromosome segregation machinery in yeast. This vector also incorporates the G418 resistance gene (KanMX) for selecting positive colonies. Vectors were introduced into yeast cells by electroporation.

For *CCW22* structure disruption, the *CCW22* ORF plus 1,000 nucleotides upstream of the start codon, containing its endogenous promoter, was cloned into the pRS vector as a wild-type control. Mutations that either disrupted or restored the *CCW22* structure were introduced based on the wild-type vector. G418-resistant clones were induced to undergo meiosis. Yeast culture (2 ml) was collected for protein extraction and Western blot analysis.

For meiosis-specific overexpression of Ded1p, we initially assessed the promoters (1,000 bp sequence upstream start codon) of 10 candidate genes using a GFP reporter system, identified by RNA-Seq and protein expression profiles. Yeast cultures (200 μL) from the same batch were sampled at various stages during meiosis, fixed with 3.7% formaldehyde, and the GFP fluorescence intensity was measured using a microplate reader. Among the candidates, the *CDC5* promoter showed the most consistent increase in GFP intensity post-Ndt80 induction (Fig. S4B) and was used for Ded1p overexpression. The coding region sequence of *DED1* was cloned into the pRS shuttle vector under the control of the *CDC5* promoter. This vector was introduced into yeast cells to assess the effects of Ded1 overexpression.

### DMS-MaP data processing

The SK1 genome sequence and annotation were sourced from a previously published dataset^15^. Adaptor sequences were removed using cutadapt^40^, and rRNA reads were depleted using a bowtie2-based alignment^41^ against rDNA sequences. For mRNA abundance quantification, DMSO library reads were mapped to the SK1 genome using STAR aligner^42^, with assigned reads counted to each gene in a strand-specific manner using featureCounts^43^. Finally, read counts were normalized by a TPM (transcripts per million) method.

For DMS-MaP, antisense reads were removed by another bowtie2-based alignment against the assembled transcriptome. The mutation counts and sequencing depth at single nucleotide resolution were parsed by ShapeMapper2^22^ using filtered fastq files as input. For stage-specific DMS reactivity, data from two replicates were merged to calculate the mutation rate, which was further normalized per transcript by the mean mutation rate of the 90–95% most reactive bases. For overall structures, DMS reactivity was calculated in a similar manner, but by pooling data from all stages.

### Protein mass spectrometry data processing

Raw mass spectrometry data were quantified using MaxQuant^44^ against the yeast proteome reference from UniProt (strain ATCC 204508 / S288c). The false discovery rate (FDR) was set at 1% at both the peptide and protein levels. Only proteins identified with at least one ‘unique/razor peptide’ in all samples were used for subsequent normalization. Total TMT intensity in each sample was scaled to 10^6^ (total normalization) following previous studies^18^.

### ORF ribosome flux and uORF ribosome footprint analysis

To quantify ribosome flux across ORFs, we divided each coding region into 50-nucleotide windows, starting from the 30th nucleotide downstream of the start codon and ending 30 nucleotides upstream of the stop codon. Ribosome footprint (A-site) counts were summed within each window, and the Gini index was calculated for windows containing more than 20 adenosine or cytosine nucleotides. The window with the highest Gini index was defined as position 0, and ribosome flux was evaluated in the immediately adjacent upstream (−1) and downstream (+1) windows.

For uORF analysis, ribosome footprints (A-site) within the −100 to +900 nucleotide region relative to the annotated start codon were normalized to a total of 1000 counts per transcript.

### RNA secondary structure modeling

DMS reactivities for adenine and cytosine with sequencing depths over 350 in both DMS-treated and DMSO-treated samples were used for structural modeling. The consensus structure model and base-pairing probabilities were predicted using SuperFold^4^, considering long-range interactions (maxPairingDist = 1000). Structural models were visualized with vaRNA^45^, and base-pair probabilities were plotted using R4RNA^46^.

### Meta-gene analysis

The average DMS reactivity was calculated for adenine and cytosine with sequencing depths over 350, based on their relative positions to the first nucleotide of the start codon or the last nucleotide of the stop codon.

### Hierarchical clustering

For RNA-Seq and ribosome profiling data, genes showing more than an 8-fold change (*P* < 0.05) in RNA abundance or ribosome footprint across all time points are log-transformed and used for subsequent clustering analysis. For quantitative proteomics data, genes showing more than 1.2-fold change (*P* < 0.05) across 4 sampled time points are used for clustering analysis. For co-hierarchical clustering of RNA helicase levels at the translation and protein levels, ribosome footprint and mass spectrometry data were first z-scored and then combined for clustering analysis. Hierarchical clustering was performed using Cluster 3.0^47^ with average clustering based on uncentered Pearson correlation. Clustering data were visualized using Java Treeview^48^.

### Differential gene expression analysis and function analysis

Fold changes in protein abundance and p-values were calculated using edgeR^49^. Proteins with a fold change over 1.2 (*P* < 0.05) were defined as differentially expressed genes. Gene Ontology enrichment analysis was conducted using g:Profiler^50^. Gene Set Enrichment Analysis was performed using GSEApy^51^, with ranked protein expression from a predefined TUMs gene set as input.

### Statistics and reproducibility

Unless specified, statistical tests were performed using the Scipy v.1.13 package in Python. Correlation analyses between Gini index and translation efficiency employed Spearman’s rank correlation. Comparisons of Spearman’s correlation coefficient between stages (Fig. 2D) were performed using the independent samples t-test. Comparisons between Gini indices were performed using the Mann-Whitney U test. Gene overlap analyses between clusters (Fig. 3D) were performed using the hypergeometric test. Statistical significance is denoted by asterisks reflecting the p-values (*P < 0.05, **P < 0.01, ***P < 0.001, ****P < 0.0001; NS, not significant).

Multiple testing corrections were applied using the False Discovery Rate (FDR) method to adjust p-values. The number of data points for each analysis is indicated in the figure legends. Unless specified, sequencing and mass spectrometry experiments were repeated twice, and other experiments were repeated at least three times with consistent results.

## Supplementary Material

1

## Data Availability

The raw sequencing data are available at the NCBI BioProject under accession number PRJNA1174166. Scripts used for sequencing data processing and analysis are accessible at the GitHub repository: https://github.com/HoNg621/StructuromeYeastMeiosis. The mass spectrometry raw data have been deposited in the ProteomeXchange Consortium member repository with accession code PXD056964. Further inquiries regarding resources and reagents should be directed to the lead contact, H.W. (wuhao@unc.edu).
